# Association of a Combination of Sarcopenia and Type 2 Diabetes with Blood Parameters, Nutrient Intake, and Physical Activity: A Nationwide Population-Based Study

**DOI:** 10.3390/nu15234955

**Published:** 2023-11-29

**Authors:** Mijin Kim, Toshiro Kobori

**Affiliations:** Institute of Food Research, National Agriculture and Food Research Organization, Tsukuba 305-8642, Japan; tkobo@affrc.go.jp

**Keywords:** diabetes, sarcopenia, blood parameters, nutritional status, physical activity, older adults

## Abstract

This study aimed to investigate the association of sarcopenia and type 2 diabetes (T2D) with blood parameters, nutrient intake, and physical activity in older Korean adults. We divided 2952 participants into four groups: sarcopenic diabetes (SD), sarcopenia alone (S), diabetes alone (D), and non-sarcopenia and non-diabetes (NSND). Sarcopenia was defined by the appendicular skeletal muscle mass index, and T2D by fasting glucose levels or ongoing treatment. Blood samples were collected after an 8-h fast. Nutrient intake was assessed using a 24-h recall; physical activity was evaluated using a questionnaire. Compared with those in the other groups, the men in the S and SD groups showed significantly lower hemoglobin and hematocrit levels; vitamin D levels in men and parathyroid hormone levels in women were significantly lower in the SD group. Total energy, protein, and carbohydrate intakes were significantly lower in the SD and S groups than those in the D and NSND groups. Physical inactivity was significantly more common in the SD group (men: odds ratio, 1.61; women: odds ratio, 2.37) than in the NSND group. A combination of sarcopenia and diabetes as well as sarcopenia alone was associated with low levels of blood parameters, poor nutrient intake, and low physical activity.

## 1. Introduction

The global prevalence of diabetes in adults (aged 20–79 years) was 10.5% (536.6 million people) in 2021. While the sex distribution was similar, the highest prevalence was observed in the 75-to-79-year age group [1]. Furthermore, a meta-analysis showed that the prevalence of sarcopenia ranges from 9.9% to 40.4% [2]. Notably, both diseases are particularly fatal to older adults because they lead to physical disabilities through complications and rapidly reduce healthy life expectancy. The decrease in skeletal muscle mass is caused by the degeneration of muscle cells, reduction of motor units and muscle fibers, hormonal changes, and deterioration of excitatory contractions [3]. Regarding nutrition, a decrease in protein intake, vitamin D insufficiency, dietary acid-base imbalance, decreased frequency and quantity of meals, and anorexia may reduce protein synthesis and promote protein degradation [4,5]. Age-related decline in skeletal muscle mass was initially termed “sarcopenia” by Rosenberg [6]. Subsequently, a diagnostic approach for sarcopenia based on appendicular skeletal muscle mass was proposed [7], and the European Working Group on Sarcopenia in Older People suggested a comprehensive diagnostic method involving reduced muscle mass, muscle strength, and/or physical function [8]. However, despite the ongoing inconsistency in the diagnostic criteria for sarcopenia, its associations with physical inactivity [9], impaired physical function [10], chronic kidney disease [11], and type 2 diabetes (T2D) [12] have been reported. Previous meta-analyses have reported that the prevalence of sarcopenia is significantly higher in individuals with diabetes (15.9%) than in those without diabetes (10.8%) [13]. Because the anabolic response of proteins to insulin declines significantly with age [14], the muscle protein synthesis response to insulin and amino acids is more impaired in older adults than in younger individuals [15]. Additionally, skeletal muscles have the largest capacity for insulin-stimulated glucose disposal in the whole body. However, age-induced skeletal muscle damage impairs glucose absorption, which results in glucose dysregulation. These further increase insulin resistance, leading to the development of T2D and accelerated skeletal muscle loss due to impaired glucose regulation [16]. This vicious cycle demonstrates the close relationship between skeletal muscle mass and glucose metabolism. Previous studies have reported the association between T2D and sarcopenia with various factors, such as low energy intake [17], decreased renal function and muscle quality [18], high serum high-sensitivity C-reactive protein [19], aging, prevalence of diabetic nephropathy and multi-morbidity [20], increased trunk fat mass and free thyroxine [21], and decreased body mass index (BMI) and physical activity [22]. Hence, the combined state of sarcopenia and T2D is expected to exacerbate the limitations of daily life in older adults. Therefore, balancing energy intake and consumption is an important countermeasure to prevent sarcopenia and T2D [12].

However, to the best of our knowledge, no study has comprehensively investigated the combined associations of sarcopenia and T2D with health status (blood parameters), nutrients (e.g., macronutrient intakes including energy), and energy expenditure (i.e., physical activity) in the daily lives of older adults. Considering the results of previous studies with similar designs, we hypothesized that the group with sarcopenia and T2D would have adverse blood parameters, poorer energy intakes, and lower physical activity levels than the other groups. This study aimed to investigate the association of a combination of sarcopenia and T2D with blood parameters, nutrient intake, and physical activity in older Korean adults.

## 2. Materials and Methods

### 2.1. Study Design and Participants

**Figure 1:**
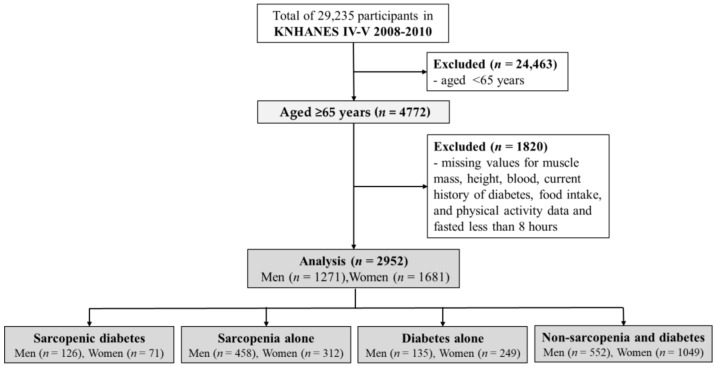
Flow diagram for the study.

This cross-sectional study utilized data collected from the Korea National Health and Nutrition Examination Survey (KNHANES) IV to V (2008–2010), conducted annually by the Korean Ministry of Health and Welfare, to investigate the health and nutritional status of the non-institutionalized civilian population of Korea [23]. A representative sample of the Korean population was selected using a multi-stage clustered probability design. From the 29,235 participants in the KNHANES, 4772 aged 65 years or older were extracted. A total of 1820 participants with missing values for muscle mass, height, blood levels, history of diabetes, food intake, physical activity, or fasting for less than 8 h were excluded. Finally, 2952 participants (43.1% men) were included in this study (Figure 1).

The KNHANES (2008-04EXP-01-C, 2009-01CON-03-2C, 2010-02CON-21-C) data used in this study were approved by the Institutional Review Board of the Korea Centers for Disease Control and Prevention, and written consent was obtained from all participants in accordance with the ethical principles of the Declaration of Helsinki.

### 2.2. Participant Characteristics

Systolic and diastolic blood pressures were measured three times on the right arm with the participant in a sitting position, after at least 5 min of rest, using a mercury sphygmomanometer (Baumanometer; Baum, Copiague, NY, USA). The average value was used in this study. Waist circumference was measured in units of 0.1 cm during expiration at the midpoint between the lowest rib and the iliac crest. BMI was calculated as body weight (kg) divided by height (m) squared. Whole-body dual-energy X-ray absorptiometry examinations were conducted using a fan-beam densitometer (Discovery-W, Hologic Inc., Bedford, MA, USA) to obtain fat mass, lean body mass, bone mass, bone area, and bone mineral density. Sleep duration (hours), drinking habits (yes or no), and smoking habits (yes or no) were assessed using self-reported questionnaires.

### 2.3. Measurement of Blood Parameters

Blood samples were collected from the participants’ veins on the morning after they had fasted for at least 8 h. Samples were immediately processed, refrigerated, and transported in cold storage to the central laboratory of Seegene Medical Foundation in Seoul, Korea. Enzyme methods were used to measure total cholesterol, high-density lipoprotein cholesterol (HDL-C), triglyceride, fasting plasma glucose (FPG), and alkaline phosphatase levels, all of which were determined using an automated analyzer (Hitachi Autometic Analyzer 7600, Tokyo, Japan). Urea nitrogen and creatinine levels were measured using a kinetic UV assay and the modified kinetic method of Jaffe, respectively, using the same analyzer. Parathyroid hormone levels were measured via chemiluminescence immunoassay using the Liaison® N-tact™ PTH Assay (DiaSorin, Stillwater, MN, USA). Hemoglobin and hematocrit levels were measured using the automated blood cell counter (Sysmex XE-2100D, Kobe, Japan). For participants who were being treated for T2D or had an FPG level of 126 mg/dL or more, glycated hemoglobin (HbA1c) was measured using high-performance liquid chromatography with automated glycohemoglobin analyzer (Tosoh HLC-723G7, Tokyo, Japan). Fasting insulin and ferritin levels were measured by immunoradiometric assay, and serum vitamin D levels were measured by radioimmunoassay using a gamma counter (1470 WIZARD, PerkinElmer, Turku, Finland).

Low-density lipoprotein-cholesterol (LDL-C) was calculated using Friedewald’s method ([Total cholesterol − HDL-C—triglyceride]/5) [24]. The homeostasis model assessment estimate of insulin resistance (HOMA-IR) value was calculated as fasting insulin (μU/mL) multiplied by FPG (mg/dL) divided by 405. The triglycerides-glucose index (TyG Index) was calculated as fasting triglyceride (mg/dL) multiplied by FPG (mg/dL) divided by two [25].

### 2.4. Assessment of Dietary Intake

The dietary intake of the participants was determined using the 24-h recall method. Daily intake of energy and nutrients, such as total energy, carbohydrates, protein, fat, crude fiber, calcium, phosphorus, iron, sodium, potassium, thiamine, riboflavin, niacin, vitamin A, and Vitamin C, was calculated using the Can-Pro 2.0 nutrient intake assessment software (The Korean Nutrition Society, Seoul, Korea) [26]. A dietary intake survey was conducted through face-to-face interviews by trained interviewers and dietitians [23].

### 2.5. Assessment of Physical Activity

Physical activity was evaluated using the Korean version of the International Physical Activity Questionnaire (IPAQ) short-form version, which consists of the frequency and duration of vigorous-intensity physical activity, moderate-intensity physical activity, and walking during the last 7 days. The total metabolic equivalent of tasks (METs) was calculated using the following formula: total METs (min/week) = ([vigorous-intensity physical activity days × vigorous-intensity physical activity minutes × 8.0 METs] + [moderate-intensity physical activity days × moderate-intensity physical activity minutes × 4.0 METs] + [walking days × walking minutes × 3.3 METs]) [27].

The World Health Organization’s (WHO) recommendation for older adults is for them to practice at least 600 METs (at least 150 min of moderate-intensity or 75 min of vigorous-intensity exercise) of physical activity per week [28]. To compare the frequency of practice by physical activity types (flexibility, resistance) and intensities (vigorous, moderate), we categorized the dependent variable as follows: 2 or more days/week for flexibility exercises represented as 1, otherwise 0; 2 or more days/week for resistance training represented as 1, otherwise 0; practicing moderate-intensity physical activity represented as 1, otherwise 0; and practicing vigorous-intensity physical activity represented as 1, otherwise 0. And to compare odds ratios (ORs) for inactivity by physical activity intensity, the dependent variable was defined as follows: not practicing vigorous physical activity, represented as 1, defined as “vigorous-intensity physical inactivity”, otherwise 0; not practicing moderate physical activity, represented as 1, defined as “moderate-intensity physical inactivity”, otherwise 0; not walking, represented as 1, defined as “Not walking”, otherwise 0; and total weekly physical activity less than 600 METs, represented as 1, otherwise 0.

### 2.6. Definitions of Sarcopenia and T2D

In this study, following the proposal of the initial diagnostic method for sarcopenia, the appendicular skeletal muscle mass index (AMI) was calculated by dividing the sum of the muscle mass (kg) in both arms and legs by the square of the height (m) [7]. Sarcopenia was defined as an AMI of less than 7.0 kg/m^2^ for men and less than 5.4 kg/m^2^ for women according to the muscle mass cutoffs of the Asian Working Group for Sarcopenia 2019 standard [29].

T2D was defined as an FPG level more than or equal to 126 mg/dL or as a person undergoing treatment (medicines, insulin injections) for diabetes after a medical diagnosis. The participants were divided into four groups: sarcopenic diabetes (SD), sarcopenia alone (S), diabetes alone (D), and non-sarcopenia and non-diabetes (NSND).

### 2.7. Statistical Analyses

In this study, owing to the non-normal distribution of characteristics, blood parameters, and nutrient intake variables among the four groups, the non-parametric Kruskal–Wallis test was used to compare inter-group differences. Drinking, smoking, and frequency of practice by physical activity type and intensity among the groups were compared using the Chi-square test. We applied the Dunn–Bonferroni correction as a post hoc test for the Kruskal–Wallis analysis to conduct multiple pairwise comparisons between each group and post hoc analysis of the Chi-square test was performed with Bonferroni correction. In the statistical analysis of nutrient intakes, we excluded total energy intake if it fell below 300 kcal or exceeded 5000 kcal, as these values were considered implausible. Additionally, binary logistic regression analysis was performed to estimate the ORs and 95% confidence intervals (CIs) for vigorous-intensity physical inactivity, moderate-intensity physical inactivity, not walking, and less than 600 METs in each group, with the NSND group as the reference group. Model 1 included age, BMI, and sleep duration as covariates, while Model 2 included the covariates from Model 1 as well as drinking and energy intake variables. All statistical analyses were performed using SPSS version 29.0 (IBM Corp., Armonk, NY, USA) and statistical significance was set at a *p*-value of less than 0.05.

## 3. Results

### 3.1. Participant Characteristics

As shown in Table 1, both the men and women in the SD and S groups were comparatively older than those in the other groups. Diastolic blood pressure was the highest in the NSND group for both men and women, while systolic blood pressure was the highest in the SD group. Body composition variables, such as waist circumference, BMI, fat mass, lean body mass, bone mass, bone area, bone mineral density, and AMI, were generally higher in the D and NSND groups than in the SD and S groups, with the D group having the highest values. Among women, sleep duration was significantly longer in the SD group than in the NSND and D groups, and drinking status was higher in the NSND group than in the S group.

### 3.2. Blood Parameters

As shown in Table 2, HDL-C levels were significantly lower in the D group than in the S and NSND groups in both men and women. In men, HDL-C levels were significantly lower in the SD and NSND groups than in the S group. Total cholesterol and LDL-C levels showed significant differences in men (*p* < 0.05); however, there were no significant differences between the groups according to post hoc analysis. Triglycerides in men were significantly higher in the SD and D groups than in the S group, and in the D group than in the SD and NSND groups. In women, triglyceride levels were significantly higher in the SD and D groups than in the S and NSND groups. Hemoglobin and hematocrit showed significant differences in both men and women; however, only in men were the SD and S groups significantly lower than the D and NSND groups, according to the post hoc analysis. Urea nitrogen showed significant differences in both men and women, but only in women was the S group significantly lower than the D group, according to the post hoc analysis. Creatinine levels were significantly lower in the S group than in the D group in men and significantly lower in the S and NSND groups than in the SD and D groups in women. Serum vitamin D levels in men were significantly lower in the SD group than in the D and NSND groups. Alkaline phosphatase levels in men were significantly higher in the SD and S groups than in the NSND group, and parathyroid hormone levels in women were significantly lower in the SD group than in the NSND group.

Furthermore, the D and SD groups had relatively higher values of diabetes-related blood parameters (insulin, HOMA-IR, TyG Index, FPG, and HbA1c), compared with the S and NSND groups.

### 3.3. Nutrient Intakes

It is worth noting that both men and women significantly differed in all variables, except for fat intake in women (Table 3). Specifically, in men, the total energy intake was significantly lower in the S group than in the D and NSND groups, and the SD group was lower than the NSND group. Protein intake was significantly lower in the SD and S groups than in the D and NSND groups. Fat intake was significantly lower in the S group than in the D and NSND groups, and carbohydrate intake was significantly lower in the SD and S groups than in the NSND group. In women, total energy, protein, and carbohydrate intakes were significantly lower in the S group than in the D and NSND groups.

Crude fiber, phosphorus, iron, potassium, thiamine, niacin, and vitamin C intakes were significantly lower in the S group than in the D and NSND groups for both men and women. Calcium, riboflavin, and vitamin A intakes were lower in the S group than in the D and NSND groups for men and significantly lower in the S group than in the D group for women. Remarkably, men in the SD group showed significantly lower intakes of nutrients, including crude fiber, phosphorus, iron, sodium, potassium, thiamine, riboflavin, niacin, and vitamin A, than those in the other groups. In contrast, in women, the only variable for which the SD group had significantly lower intake levels than the other groups was sodium intake.

### 3.4. Physical Activities

Table 4 and Table 5 present the results of the physical activity analysis.

In Table 4, for men, the rate of practicing flexibility exercises for more than 2 days a week was significantly lower in the S group (34.9%) compared with the NSND group (43.1%). Similarly, the practice rate of resistance training of more than 2 days a week was significantly lower in the S group (19.0%) compared with the D (31.9%) and NSND groups (28.1%). Additionally, the practice rate of vigorous-intensity physical activity for more than 75 min a week was lower in the S group (16.2%) compared with the D group (29.6%) and significantly lower in the SD group (8.7%) compared with the D (29.6%) and NSND groups (19.4%). On the other hand, for women, the rate of practicing moderate-intensity physical activity for more than 2 days a week was significantly lower in the SD group (7.0%) compared with the NSND group (14.2%).

In Table 5, for vigorous-intensity physical inactivity, the SD group (Model 1: OR, 2.78; 95% CI, 1.47–5.33, Model 2: OR, 2.68; 95% CI, 1.42–5.19) had a significantly higher risk rate than did the NSND group in men; however, there was no difference between the groups in women. In moderate-intensity physical inactivity, the SD (Model 1: OR, 1.70; 95% CI, 1.11–2.66, Model 2: OR, 1.61; 95% CI, 1.06–2.55) and S (Model 1: OR, 1.57; 95% CI, 1.12–2.09, Model 2: OR, 1.49; 95% CI, 1.06–1.99) groups had significantly higher risk rates than did the NSND group in men. In women, the SD (Model 1: OR, 3.63; 95% CI, 1.93–7.62, Model 2: OR, 3.58; 95% CI, 1.91–7.56) and S (Model 1: OR, 1.40; 95% CI, 1.02–1.84, Model 2: OR, 1.37; 95% CI, 1.01–1.81) groups also showed significantly higher risk rates than did the NSND group. In not walking, the D group (Model 1: OR, 0.51; 95% CI, 0.30–1.03, Model 2: OR, 0.50; 95% CI, 0.30–1.03) in men had a higher practice rate than did the NSND group. In the unadjusted model for women, the OR for not walking was 1.99 (95% CI: 1.34–3.54) in the SD group compared with the NSND group. In the adjusted models, both Model 1 (OR: 1.69; 95% CI: 1.14–3.18; *p* = 0.053) and Model 2 (OR: 1.70; 95% CI: 1.14–3.19; *p* = 0.051) showed a significant trend. In less than 600 METs (min/week), the SD group in men (Model 1: OR, 1.69; 95% CI, 1.14–2.71, Model 2: OR, 1.61; 95% CI, 1.08–2.58) and women (Model 1: OR, 2.41; 95% CI, 1.53–4.15, Model 2: OR, 2.37; 95% CI, 1.50–4.07) had significantly higher risk rates than did the NSND group.

## 4. Discussion

The present study found that the combination of sarcopenia and T2D in older adults was associated with low levels of blood parameters (hemoglobin, hematocrit, vitamin D, and parathyroid hormone), poor nutrient intake (including protein and carbohydrate intake), and low physical activity (vigorous and moderate-intensity activity, walking, <600 METs). The energy intake within the body and energy expended through activity are associated with temporary and long-term fluctuations in the levels of blood parameters. Given that low energy intake and low physical activity are associated with lower blood parameter values for hemoglobin, hematocrit, vitamin D, and parathyroid hormone, these results may imply a relationship between the dependent variables.

Increased triglyceride levels can cause dyslipidemia associated with insulin resistance and T2D [30]; consequently, T2D is likely to increase the risk of developing cardiovascular diseases [31]. Additionally, a previous study found that skeletal muscle-associated triglyceride level is inversely proportional to insulin sensitivity [32]. Similarly, in this study, triglyceride levels were significantly higher in the groups with diabetes, namely the SD (median: 120.0 mg/dL in men,151.0 mg/dL in women) and D groups (median: 147.0 mg/dL in men, 125.0 mg/dL in women), compared with the S and NSND groups. Furthermore, the TyG index, which is calculated using triglycerides and FPG levels, has been suggested as a useful surrogate marker for insulin resistance [33]. Systematic reviews and meta-analyses have reported its usefulness in diagnosing metabolic syndrome [34]. High blood lipid levels observed in obesity and metabolic syndrome cause lipotoxicity and ectopic fat deposition in other organs, resulting in changes in skeletal muscle composition, insulin resistance, and impaired glucose metabolism [35]. In this study, the D and SD groups had higher levels of diabetes-related factors (insulin, HOMA-IR, TyG Index, FPG, and HbA1c) and obesity-related factors (triglycerides, waist circumference, BMI, and fat mass) than did the S group. The high values of obesity-related variables suggest that the possibility of obesity is included. In particular, the SD group was implicated in sarcopenic obesity, in which sarcopenia and obesity coexist. According to systematic reviews and meta-analyses, the prevalence of sarcopenia is associated with approximately 40 to 45% of overweight and obese individuals, and sarcopenic obesity increases the risk of T2D by 38% compared with obesity alone [36]. Therefore, there appears to be a bidirectional interaction between T2D, obesity, and sarcopenia, and future longitudinal studies are needed to reveal a causal relationship between them.

Among the blood parameters, reduced hemoglobin and hematocrit levels are associated with conditions such as anemia, polycythemia, cardiovascular disease, Crohn’s disease, iron deficiency, inadequate protein intake, and an increased risk of mortality related to malnutrition [18,37,38]. A previous study indicated a positive correlation between muscle mass, hemoglobin, and hematocrit in patients with T2D [37]. This suggests a potential connection between anemia in patients with T2D and the presence of sarcopenia [38]. Another study reported that men with sarcopenia and T2D had significantly lower levels of hemoglobin and hematocrit [18]. Similarly, the present study also found that men in the S and SD groups had lower hemoglobin and hematocrit levels than those in the other groups. In addition, the nutritional intake status was lowest in the S and SD groups among men and in the S group among women. Furthermore, among older adults, the risk of sarcopenia was higher in individuals with poor nutritional status (OR: 4.07) or T2D (OR: 5.15) compared with that of those without these conditions [39]. Both sarcopenia and T2D are strongly correlated with anemia (characterized by low hemoglobin and hematocrit levels) and poor nutritional intake. Consequently, enhancing nutritional intake is crucial for addressing anemia, sarcopenia, and T2D. In addition, vitamin D is necessary for improving sarcopenia because it helps strengthen bones and promotes protein synthesis along with calcium in the musculoskeletal system. Low vitamin D levels are associated with musculoskeletal system disorders, microbial diseases, cardiovascular mortality, and metabolic diseases. Furthermore, vitamin D deficiency adversely affects insulin synthesis and secretion, increasing the risk of developing T2D [40]. Therefore, maintaining 25-hydroxyvitamin D (25(OH)D) levels of more than 30 ng/mL is recommended [41]. The criterion for 25(OH)D deficiency is less than 20 ng/mL, and between 21 and 29 ng/mL is defined as vitamin D insufficiency [42]. In this study, the 25(OH)D level in men was significantly lower in the SD group (median: 17.5 ng/mL) compared with that of the NSND group (median: 22.7 ng/mL). Applying the cutoff values from previous studies, it was evident that all four groups of men and women in this study had low 25(OH)D levels. Low vitamin D and calcium intake can lead to secondary hyperparathyroidism, high bone turnover, bone loss, fractures, and falls [43,44]. Moreover, hyperparathyroidism is associated with weight loss and impaired muscle function [45]. A previous study indicated that parathyroid hormone levels are linked to decreased insulin release and sensitivity [46]. Furthermore, high levels of parathyroid hormones are associated with a two to four-times higher incidence of diabetes [46]. In this study, the SD group in women had lower parathyroid hormone levels than those in the NSND group. In contrast, a previous study showed no differences in 25(OH)D and parathyroid hormone levels between groups with and without sarcopenia in T2D [21]. As changes in parathyroid hormone levels related to 25(OH)D levels can have contradictory effects on muscle and bone function as well as glucose metabolism, a more in-depth investigation is needed to understand their effects on the complications of sarcopenia and diabetes.

The aging of skeletal muscle cells decreases their capacity for glucose disposal; therefore, older adults with sarcopenia and diabetes need to pay attention to their carbohydrate and protein intake. The WHO recommends a daily protein intake of 0.8 g/kg of body weight (kg)/day (d) for all adults [47]. However, to maintain and improve muscle function in older adults, it is recommended that they consume more protein than younger individuals do. When it comes to protein intake, the PROT-AGE Study, focusing on protein requirements for older adults, suggests 1.0–1.2 g/kg/d [48]. The American Diabetes Association recommends 1.0–1.5 g/kg/d for individuals with diabetes without kidney disease [49], and a nutritional review study on sarcopenia proposed 75–90 g/d [50]. In this study, the daily total protein intake was low in the SD (median: 52.0 g) and S (median: 53.0 g) groups for men and in the S group (median: 37.0 g) for women. Hence, there is a need to increase protein intake, as all groups were shown to consume less protein than what was recommended by previous studies.

Regarding carbohydrate intake, the WHO recommends aiming for approximately 55–75% of the total daily energy intake for adults and limiting free sugars to less than 10%; moreover, they encourage the consumption of non-starch polysaccharides from sources such as whole-grain cereals, fruits, vegetables, and beans rather than simple carbohydrates [51]. Furthermore, dietary fibers, which are non-starch polysaccharides, prevent sharp increases in blood glucose levels caused by carbohydrates and lower cholesterol levels. Therefore, it is necessary to consider not only the total carbohydrate intake but also factors such as the type of sugar, form and nature of food, cooking, and processing methods, as they all influence glycemic responses [49].

Additionally, carbohydrate intake after fasting can contribute to increased sodium retention in the kidneys, along with hyperinsulinemia, which leads to obesity and hypertension [52]. Therefore, patients with diabetes are required to regulate their dietary intake of sodium because of concerns about complications related to hypertension. To prevent hypertension, it is recommended that sodium chloride (salt) consumption is less than 6000 mg/d [49]. Sodium plays crucial roles in regulating fluid balance, transmitting nerve impulses, maintaining muscle function, and controlling blood pressure. It has also been reported that insulin-mediated changes are related to sodium transport in the kidneys [52]. In this study, both men and women in the SD group had the lowest levels of sodium intake. Sodium intake was high in the D and NSND groups but did not exceed the recommended daily intake (6000 mg/day). A previous study reported that the total energy intake was lower in individuals with T2D and sarcopenia than in those with T2D without sarcopenia. Although there were no differences between the two groups in terms of protein and carbohydrate intakes, fat intake was lower in those with T2D and sarcopenia [17]. However, the findings of this study contradict those of previous studies. Total energy, protein, and carbohydrate intakes were the lowest in the S and SD groups in men and in the S group in women. Moreover, the fat intake was lower in men in the S group. These results suggest that nutrient intake may be more significantly influenced by sarcopenia than diabetes alone.

Older adults with T2D who are overweight may require dietary restrictions aimed at weight loss. However, for older adults with T2D who are underweight, such as those with sarcopenia in long-term care facilities, it may be better to consider maintaining a consistent carbohydrate intake in terms of quantity and timing. Furthermore, instead of dietary restrictions, the focus should be on protein intake, physical activity, and medication adjustments [49]. However, while T2D is commonly treated with antidiabetic medications to regulate blood glucose levels, hormonal therapy for muscle enhancement is rarely prescribed in cases of sarcopenia, owing to its potential side effects [12]. Therefore, the most effective approach for simultaneously preventing and improving low muscle mass, insulin resistance, and insulin sensitivity is a combination of adequate nutrient intake and high levels of physical activity. Interestingly, in this study, the combination of sarcopenia and T2D showed significantly negative results not only in nutrient intake but also in physical activity. Physical inactivity has been reported to not only affect conditions such as sarcopenia [9], but also aging and diseases, including diabetes [28,53]. Prolonged physical inactivity owing to age-related diseases or hospitalization leads to a rapid decline in skeletal muscle and physical function, resulting in the onset of sarcopenia [54]. The effectiveness of health promotion through physical activity varies according to the dose-response relationship; therefore, it is necessary to set exercise intensity according to its desired purpose. According to the guidelines recommended by the American College of Sports Medicine and the American Heart Association [55], healthy older adults should practice aerobic activity of moderate-intensity for at least 30 min, 5 days/week, or vigorous intensity for at least 20 min, 3 days/week. Additionally, for muscle strengthening, they recommend performing resistance training at least 2 days/week, with 8–12 repetitions of 8–10 exercises targeting major muscle groups. The WHO [28] recommends at least 150–300 min of moderate-intensity or at least 75–150 min of vigorous-intensity aerobic physical activity throughout the week for adults and older adults with chronic conditions. In addition, resistance training should be performed at least 2 days/week at moderate or high intensity to reduce sedentary behavior. In this study, the rate of practicing flexibility exercises and resistance training at least 2 days/week was the lowest in those with sarcopenia alone in men (Table 4). Additionally, compared with the reference group, the combination of sarcopenia and diabetes was associated with a more than doubling of the risk of vigorous-intensity physical inactivity in men in all models of Table 5 (OR: 2.87 in crude model, OR: 2.78 in model 1, OR: 2.68 in model 2). On the other hand, all women participants had a low rate of practicing vigorous-intensity physical activity, so there was no difference between the groups. In addition, the risk rate of moderate-intensity physical inactivity was significantly higher in the combination of sarcopenia and diabetes. It was more than 1.5 times higher in men and 3.5 times higher in women. Furthermore, sarcopenia alone was associated with a risk rate of more than 1.3 times for both men and women compared with the reference group; however, diabetes alone was not. In particular, the risk rate of physical inactivity of less than 600 METs, calculated as the minimum intensity and time, was high in the combination of sarcopenia and diabetes for both men (over OR 1.6 in all models) and women (over OR 2.3 in all models). Taken together, these results suggest that the combination of sarcopenia and diabetes has the greatest impact on vigorous- and moderate-intensity inactivity in men and moderate-intensity inactivity in women, and that sarcopenia alone is more strongly associated with physical inactivity than diabetes alone.

This study has certain strengths. It used a large-scale epidemiological survey (KNHANES) conducted by the Ministry of Korea, and the results were analyzed using data obtained through rigorous measurements and reviews by experts. However, this study also had several limitations. First, this study had sampling bias and an unstandardized definition for sarcopenia diagnosis. As the study population was limited to older Korean adults, the results may not be representative of older adults worldwide. Additionally, sarcopenia was evaluated solely on the basis of the loss of muscle mass, which was the initial diagnostic method, because data on muscle strength and physical performance were not available in the KNHANES from 2008 to 2010, when muscle mass was measured. The prevalence of sarcopenia varies widely, ranging from 9.9 to 40.4%, depending on the definition and diagnostic criteria used [2]. Because results may vary depending on the criteria used to diagnose sarcopenia, future studies should consider evaluating the use of a comprehensive sarcopenia diagnostic method. Second, since the data on nutritional intake were estimated from the 24-h recall, they may not reflect all the information about daily changes in dietary intake. In addition, the 24-h recall method for dietary intake assessment may have recall bias, and although face-to-face interviews were conducted with participants by experts, it may not be possible to entirely eliminate the potential for memory errors, particularly among the older adults compared with younger adults. Therefore, in this study, participants were asked to compare their food intake over 24 h with their usual intake, with response options (Ate more than usual, Ate the same as usual, Ate less than usual, Table 3). More than 84% of participants in each group reported eating the same amount as usual. These results indicate that, in contrast to younger individuals who may experience significant variations in daily food intake due to gatherings, dining out, and overeating, older adults generally maintain more consistent eating patterns. However, in future studies, it is necessary to investigate food intake frequency and quantity through more accurate measurement methods. Third, the IPAQ, which was used to evaluate the amount of physical activity, consisted of subjective data based on a self-reported questionnaire. However, the IPAQ has been widely used worldwide, and its reliability and validity have been recognized [56]. Nevertheless, self-reported questionnaires themselves may carry the potential for response bias. In questionnaires assessing physical activity, including the IPAQ, participants are allowed to provide dual responses for each type of activity (vigorous-intensity, moderate-intensity, and low-intensity (walking)). Therefore, in this study, the classification of physical inactivity may include the possibility of performing in another intensity of activity. Additionally, since the IPAQ-short form has fewer questions compared with the IPAQ-long form, physical activity levels may be underestimated. Therefore, in future research, it is necessary to consider using the IPAQ-long form or objective data measured by devices such as actigraphy. Fourth, because the data in this study were obtained through a cross-sectional design, the causal relationship between sarcopenia and T2D cannot be explained. Therefore, a causal relationship analysis of the development of sarcopenia and diabetes should be conducted through a longitudinal study in future research.

## 5. Conclusions

In this study, it has been demonstrated that the combination of sarcopenia and diabetes is associated with low levels of blood parameters (hemoglobin, hematocrit, vitamin D, and parathyroid hormone), poor nutritional status (including protein and carbohydrate intake), and low physical activity (vigorous and moderate-intensity activity, walking, <600 METs). In addition, sarcopenia alone has been shown to have been more negatively associated with blood parameters, nutrient intake, and physical activity than diabetes alone. The findings of this study may serve as foundational data for the interpretation of longitudinal studies aimed at elucidating causal relationships between these two factors. Moreover, our study may contribute to the development of intervention programs emphasizing balanced energy intake and consumption with the goal of preventing and improving the comorbid conditions of sarcopenia and diabetes.

## Figures and Tables

**Table 1:** Participant characteristics by sarcopenia and diabetes groups.

Men										
Variables (Unit)	Sarcopenic Diabetes ^a^ (*n* = 126)		Sarcopenia Alone ^b^ (*n* = 458)		Diabetes Alone ^c^ (*n* = 135)		Non-Sarcopenia and Non-Diabetes ^d^ (*n* = 552)		K-W Test	
	Median	(IQR: Q1–Q3)	Median	(IQR: Q1–Q3)	Median	(IQR: Q1–Q3)	Median	(IQR: Q1–Q3)	*H*-Value	*p*-Value
Age (year)	72.0	(69.0–75.0) ^c,d^	73.0	(69.0–76.0) ^c,d^	69.0	(67.0–73.0) ^a,b^	70.0	(67.0–74.0) ^a,b^	66.7	<0.01
SBP (mmHg)	131.0	(114.0–145.0)	129.0	(117.0–141.0)	130.0	(121.0–141.0)	129.0	(118.0–141.0)	1.5	0.69
DBP (mmHg)	74.0	(69.0–82.0) ^d^	77.0	(70.0–82.0) ^d^	77.0	(70.0–83.0)	79.0	(70.0–84.0) ^a,b^	15.9	<0.01
WC (cm)	84.2	(79.1–88.7) ^b,c,d^	79.3	(73.4–85.3) ^a,c,d^	91.7	(85.2–95.0) ^a,b,d^	87.6	(82.7–92.3) ^a,b,c^	279.5	<0.01
BMI (kg/m^2^)	22.2	(20.9–23.6) ^b,c,d^	21.0	(19.4–22.5) ^a,c,d^	25.4	(23.5–26.9) ^a,b,d^	24.3	(22.9–25.9) ^a,b,c^	479.7	<0.01
Fat mass (kg)	15.3	(11.7–18.1) ^b^	12.2	(9.0–15.7) ^a,c,d^	15.6	(13.3–19.9) ^b,d^	15.0	(11.9–18.2) ^b,c^	101.7	<0.01
LBM (kg)	43.4	(39.6–46.1) ^b,c,d^	41.5	(38.8–44.0) ^a,c,d^	50.0	(46.6–53.7) ^a,b,d^	48.1	(45.3–51.8) ^a,b,c^	568.5	<0.01
Bone mass (g)	2261.4	(2013.0–2563.3) ^b,c,d^	2142.2	(1927.2–2363.3) ^a,c,d^	2480.7	(2273.9–2748.7) ^a,b^	2410.9	(2194.5–2652.6) ^a,b^	160.7	<0.01
Bone area (cm^2^)	2005.3	(1884.7–2112.0) ^b,c,d^	1960.6	(1830.1–2057.9) ^a,c,d^	2123.5	(2011.9–2234.1) ^a,b^	2093.2	(1996.7–2196.1) ^a,b^	221.2	<0.01
BMD (g/cm^2^)	1.13	(1.0–1.2) ^c^	1.10	(1.0–1.2) ^c,d^	1.16	(1.1–1.2) ^a,b^	1.15	(1.1–1.2) ^b^	59.4	<0.01
AMI (kg/cm^2^)	6.50	(6.1–6.8) ^c,d^	6.55	(6.2–6.8) ^c,d^	7.61	(7.3–8.1) ^a,b^	7.62	(7.3–8.0) ^a,b^	946.5	<0.01
Sleep duration (hour)	7.0	(6.0–8.0)	7.0	(6.0–8.0)	7.0	(6.0–8.0)	7.0	(6.0–8.0)	7.1	0.07
^†^ Drinking (yes, n(%))	77 (61.1)		282 (61.6) ^d^		94 (69.6)		390 (70.7) ^b^			<0.01
^†^ Smoking (yes, n(%))	70 (55.6)		280 (61.1)		80 (59.3)		344 (62.3)			0.54
**Women**										
**Variables (Unit)**	**Sarcopenic diabetes ^a^** **(*n* = 71)**		**Sarcopenia alone ^b^** **(*n* = 312)**		**Diabetes alone ^c^** **(*n* = 249)**		**Non-sarcopenia** **and non-diabetes ^d^** **(*n* = 1049)**		**K-W test**	
	**Median**	**(IQR: Q1–Q3)**	**Median**	**(IQR: Q1–Q3)**	**Median**	**(IQR: Q1–Q3)**	**Median**	**(IQR: Q1–Q3)**	***H*-value**	***p*-value**
Age (year)	73.0	(68.0–77.0) ^c^	72.0	(68.0–76.0) ^c,d^	70.0	(67.0–75.0) ^a,b^	71.0	(68.0–75.0) ^b^	23.5	<0.01
SBP (mmHg)	137.5	(122.5–150.5) ^b^	131.0	(118.0–142.0) ^a^	133.0	(120.0–144.0)	132.0	(120.0–144.0)	8.4	0.04
DBP (mmHg)	76.0	(69.0–82.0)	75.0	(70.0–82.0) ^c^	76.0	(70.0–82.0)	79.0	(71.0–85.0) ^b^	18.3	<0.01
WC (cm)	81.2	(74.3–85.8) ^b,c^	77.1	(71.1–84.2) ^a,c,d^	89.4	(83.2–95.2) ^a,b,d^	83.6	(77.4–89.9) ^b,c^	228.6	<0.01
BMI (kg/m^2^)	22.4	(20.6–24.0) ^c,d^	21.4	(19.6–23.2) ^c,d^	25.8	(23.8–27.9) ^a,b,d^	24.5	(22.5–26.5) ^a,b,c^	324.2	<0.01
Fat mass (kg)	18.3	(14.5–22.2) ^c^	16.7	(13.3–20.1) ^c,d^	20.8	(17.6–24.7) ^a,b,d^	19.0	(15.2–22.7) ^b,c^	87.8	<0.01
LBM (kg)	30.7	(28.9–32.4) ^c,d^	29.9	(28.2–31.5) ^c,d^	36.3	(34.2–39.7) ^a,b,d^	34.5	(32.4–37.0) ^a,b,c^	560.7	<0.01
Bone mass (g)	1524.4	(1299.3–1727.5) ^c^	1437.1	(1252.9–1646.0) ^c,d^	1676.9	(1484.6–1842.5) ^a,b,d^	1580.8	(1406.2–1774.2) ^b,c^	91.2	<0.01
Bone area (cm^2^)	1557.3	(1440.9–1678.9) ^c,d^	1552.7	(1450.5–1676.9) ^c,d^	1706.7	(1595.7–1820.3) ^a,b,d^	1666.0	(1562.4–1768.3) ^a,b,c^	148.1	<0.01
BMD (g/cm^2^)	0.96	(0.9–1.0)	0.92	(0.9–1.0) ^c,d^	0.97	(0.9–1.0) ^b,d^	0.94	(0.9–1.0) ^b,c^	30.4	<0.01
AMI (kg/cm^2^)	5.15	(4.9–5.3) ^c,d^	5.15	(4.9–5.3) ^c,d^	6.22	(5.9–6.6) ^a,b^	6.07	(5.8–6.4) ^a,b^	896.6	<0.01
Sleep duration (hour)	7.0	(6.0–8.0) ^c,d^	6.0	(5.0–8.0)	6.0	(5.0–8.0) ^a^	6.0	(5.0–7.0) ^a^	10.5	0.01
^†^ Drinking (yes, *n* (%))	20 (28.2)		94 (30.1)		83 (33.3)		374 (35.7)			0.21
^†^ Smoking (yes, *n* (%))	7 (9.9)		36 (11.5)		20 (8.0)		84 (8.0)			0.26

Note: *p*-values from Kruskal–Wallis (K-W) test and ^†^ Chi-square test. Significantly different (*p* < 0.05) from the: ^a^ Sarcopenic diabetes group, ^b^ Sarcopenia alone group, ^c^ Diabetes alone group, and ^d^ Non-sarcopenia and non-diabetes group. Post hoc tests for multiple pairwise comparisons between each group utilized Kruskal–Wallis analysis with Dunn–Bonferroni correction and Chi-square test with Bonferroni correction. IQR: interquartile range, Q: quartile (Q1: 25th percentile, Q3: 75th percentile), SBP: systolic blood pressure, DBP: diastolic blood pressure, BMI: body mass index, AMI: appendicular skeletal muscle mass index, *n*: number of participants.

**Table 2:** Comparison in blood biochemical parameters among the sarcopenia and diabetes groups.

Men										
Variables (Unit)	Sarcopenic Diabetes ^a^ (*n* = 126)		Sarcopenia Alone ^b^ (*n* = 458)		Diabetes Alone ^c^ (*n* = 135)		Non-Sarcopenia and Non-Diabetes ^d^ (*n* = 552)		K-W Test	
	Median	(IQR: Q1–Q3)	Median	(IQR: Q1–Q3)	Median	(IQR: Q1–Q3)	Median	(IQR: Q–Q3)	*H*-Value	*p*-Value
TC (mg/dL)	172.0	(148.8–195.3)	176.0	(155.0–201.0)	172.0	(153.0–198.0)	182.0	(158.0–203.0)	7.9	0.05
HDL-C (mg/dL)	41.7	(35.6–48.9) ^b^	45.2	(38.2–53.9) ^a,c,d^	39.1	(33.0–45.2) ^b,d^	42.6	(36.5–49.5) ^b,c^	43.8	<0.01
LDL-C (mg/dL)	101.7	(78.0–124.3)	106.3	(87.5–131.0)	103.7	(82.9–129.1)	110.7	(88.9–130.8)	9.6	0.02
Triglyceride (mg/dL)	120.0	(83.0–184.5) ^c^	107.0	(76.0–154.3) ^c,d^	147.0	(111.0–215.0) ^a,b,d^	119.5	(84.0–168.0) ^b,c^	38.2	<0.01
Hemoglobin (g/dL)	14.0	(13.2–15.3) ^c,d^	14.3	(13.4–15.1) ^c,d^	14.7	(13.9–15.5) ^a,b^	14.8	(14.0–15.4) ^a,b^	46.7	<0.01
Hematocrit (%)	41.6	(39.1–44.8) ^c,d^	42.5	(40.0–44.6) ^c,d^	43.4	(41.1–45.6) ^a,b^	43.5	(41.5–45.5 )^a,b^	39.3	<0.01
Ferritin (ng/mL)	97.2	(58.8–168.1)	88.1	(52.4–150.1)	98.6	(57.4–184.0)	89.5	(53.5–157.4)	4.6	0.20
Urea nitrogen (mg/dL)	17.0	(14.0–21.0)	16.0	(14.0–19.0)	18.0	(14.0–21.0)	16.0	(14.0–19.0)	8.3	0.04
Creatinine (mg/dL)	1.00	(0.8–1.1)	0.90	(0.8–1.0) ^c^	1.00	(0.9–1.1) ^b^	0.93	(0.8–1.1)	16.9	<0.01
Vitamin D (ng/mL)	17.5	(13.8–23.2) ^b,d^	21.8	(16.5–27.7) ^a^	20.3	(14.9–26.5)	22.7	(17.8–27.5) ^a^	33.7	<0.01
PH (pg/mL)	64.3	(50.4–79.1)	64.1	(50.7–81.1)	63.8	(47.8–78.7)	61.4	(49.7–77.4)	3.3	0.35
Insulin (μU/mL)	9.1	(6.7–11.6) ^b^	7.4	(5.8–9.7) ^a,c,d^	9.0	(7.7–13.2) ^b,d^	8.4	(6.7–11.5) ^b,c^	69.0	<0.01
HOMA-IR	2.98	(2.2–4.2) ^b^	1.75	(1.3–2.3) ^a,c,d^	3.13	(2.3–4.5) ^b,d^	2.04	(1.6–2.8) ^a,b,c^	215.7	<0.01
TyG Index	4.80	(4.6–5.1) ^b,c,d^	4.61	(4.4–4.8) ^a,c,d^	4.93	(4.8–5.2) ^a,b,d^	4.67	(4.5–4.9) ^a,b,c^	130.0	<0.01
FPG (mg/dL)	131.0	(113.8–150.5) ^b,d^	94.0	(88.0–102.0) ^a,c^	129.0	(108.0–148.0) ^b,d^	96.0	(90.0–103.8) ^a,c^	360.7	<0.01
HbA1C (%)	6.8	(6.2–8.1)			6.8	(6.2–7.6)				
**Women**										
**Variables (Unit)**	**Sarcopenic diabetes ^a^** **(*n* = 71)**		**Sarcopenia alone ^b^** **(*n* = 312)**		**Diabetes alone ^c^** **(*n* = 249)**		**Non-sarcopenia** **Band non-diabetes ^d^** **(*n* = 1049)**		**K-W test**	
	**Median**	**(IQR: Q1-Q3)**	**Median**	**(IQR: Q1-Q3)**	**Median**	**(IQR: Q1-Q3)**	**Median**	**(IQR: Q1-Q3)**	***H*-value**	***p*-value**
TC (mg/dL)	197.0	(161.0–225.0)	198.5	(180.0–222.0)	195.0	(166.0–220.0)	199.0	(176.0–223.0)	4.4	0.22
HDL-C (mg/dL)	44.3	(38.2–51.3)	46.1	(39.1–53.0) ^c^	44.3	(36.5–50.4) ^b,d^	45.2	(39.1–52.2) ^c^	10.0	0.02
LDL-C (mg/dL)	116.7	(87.8–157.5)	126.2	(105.9–144.8)	123.3	(93.5–144.4)	125.8	(104.3–147.3)	4.5	0.21
Triglyceride (mg/dL)	151.0	(106.0–219.0) ^b,d^	125.0	(86.0–175.0) ^a,c^	135.0	(99.0–191.0) ^b,d^	122.0	(88.0–177.0) ^a,c^	18.5	<0.01
Hemoglobin (g/dL)	12.8	(12.2–13.6)	12.9	(12.1–13.6)	13.1	(12.3–13.8)	13.0	(12.3–13.7)	9.5	0.02
Hematocrit (%)	38.4	(36.5–40.4)	38.7	(36.5–40.8)	39.4	(36.9–41.3)	39.3	(37.2–41.1)	8.8	0.03
Ferritin (ng/mL)	50.1	(26.9–89.2)	58.9	(38.2–90.2)	62.8	(36.6–94.1)	60.5	(37.5–90.7)	3.0	0.40
Urea nitrogen (mg/dL)	15.0	(12.0–19.0)	15.0	(12.0–18.0) ^c^	16.0	(13.0–19.5) ^b^	15.0	(13.0–19.0)	10.3	0.02
Creatinine (mg/dL)	0.80	(0.7–0.9) ^b,d^	0.70	(0.6–0.8) ^a,c^	0.77	(0.7–0.9) ^b,d^	0.70	(0.6–0.8) ^a,c^	28.6	<0.01
Vitamin D (ng/mL)	17.4	(14.6–22.5)	16.9	(12.8–23.1)	17.4	(13.7–23.4)	18.5	(13.7–24.4)	6.2	0.10
PH (pg/mL)	61.3	(46.0–74.9) ^d^	63.7	(49.8–84.4)	66.7	(50.7–84.9)	66.9	(53.0–85.3) ^a^	8.9	0.03
Insulin (μU/mL)	10.1	(7.1–13.5) ^b^	8.7	(6.9–10.7) ^a,c,d^	11.4	(8.7–15.3) ^b,d^	9.2	(7.3–11.9) ^b,c^	75.4	<0.01
HOMA-IR	3.31	(2.2–4.3) ^b,d^	2.00	(1.6–2.5) ^a,c,d^	3.67	(2.7–5.0) ^b,d^	2.15	(1.6–2.8) ^a,b,c^	271.4	<0.01
TyG Index	4.93	(4.7–5.1) ^b,d^	4.69	(4.5–4.9) ^a,c^	4.90	(4.7–5.1) ^b,d^	4.68	(4.5–4.9) ^a,c^	143.9	<0.01
FPG (mg/dL)	130.0	(98.0–159.0) ^b,d^	93.0	(88.0–100.8) ^a,c^	129.0	(113.5–144.5) ^b,d^	94.0	(89.0–101.0) ^a,c^	479.1	<0.01
HbA1 C (%)	6.9	(6.2–7.9)			6.9	(6.4–7.6)				

Note: *p*-values are from the Kruskal–Wallis (K-W) test. Significantly different (*p* < 0.05) from the: ^a^ Sarcopenic diabetes group, ^b^ Sarcopenia alone group, ^c^ Diabetes alone group, and ^d^ Non-sarcopenia and non-diabetes group. Dunn–Bonferroni post hoc analyses were used to identify any group difference. IQR: interquartile range, Q: quartile (Q1: 25th percentile, Q3: 75th percentile), TC: total cholesterol, HDL-C: high density lipoprotein cholesterol, LDL-C: low density lipoprotein cholesterol, PH: parathyroid hormone, HOMA-IR: homeostatic model assessment for insulin resistance, TyG Index: triglyceride-glucose index, FPG: fasting plasma glucose, HbA1c: glycated hemoglobin (HbA1c was measured only for those who were being treated for diabetes or whose FPG was greater than or equal to126 mg/dL), *n*: number of participants.

**Table 3:** Comparison of nutrient intakes among the sarcopenia and diabetes groups.

Men										
Variables (Unit)	Sarcopenic Diabetes ^a^ (*n* = 126)		Sarcopenia Alone ^b^ (*n* = 458)		Diabetes Alone ^c^ (*n* = 135)		Non-Sarcopenia and Non-Diabetes ^d^ (*n* = 552)		K-W Test	
	Median	(IQR: Q1–Q3)	Median	(IQR: Q1–Q3)	Median	(IQR: Q1–Q3)	Median	(IQR: Q1–Q3)	*H*-Value	*p*-Value
Total energy (kcal)	1680.2	(1349.3–2142.9) ^d^	1729.8	(1378.7–2071.3) ^c,d^	1919.5	(1395.1–2305.4) ^b^	1832.5	(1550.2–2336.6) ^a,b^	32.5	<0.01
Carbohydrate (g)	298.4	(242.9–354.5) ^d^	304.3	(250.7–378.7) ^d^	333.4	(248.2–395.5)	325.8	(274.3–399.8) ^a,b^	23.0	<0.01
Protein (g)	52.0	(37.6–73.2) ^c,d^	53.0	(40.2–69.5) ^c,d^	65.1	(44.5–82.8) ^a,b^	60.5	(46.0–83.2) ^a,b^	34.6	<0.01
Fat (g)	21.4	(11.8–32.8)	17.8	(11.4–29.3) ^c,d^	25.1	(13.9–41.4) ^b^	21.8	(13.8–37.1) ^b^	25.8	<0.01
Crude Fiber (g)	6.52	(4.1–9.0) ^c^	6.15	(4.3–8.8) ^c,d^	8.10	(5.6–11.0) ^a,b^	6.75	(4.7–10.1) ^b^	23.2	<0.01
Calcium (mg)	364.8	(213.1–600.5)	354.1	(220.4–535.1) ^c,d^	453.7	(283.8–699.6) ^b^	407.2	(283.1–609.6) ^b^	23.0	<0.01
Phosphorus (mg)	990.1	(707.1–1324.6) ^c,d^	976.1	(768.0–1251.2) ^c,d^	1138.1	(886.9–1434.0) ^a,b^	1117.2	(875.4–1443.1) ^a,b^	37.4	<0.01
Iron (mg)	11.2	(6.7–17.2) ^c^	10.7	(7.0–16.9) ^c,d^	14.4	(8.9–19.3) ^a,b^	12.3	(8.3–18.7) ^b^	23.3	<0.01
Sodium (mg)	3668.3	(2247.9–5374.8) ^c,d^	4044.6	(2599.8–5681.0) ^d^	4213.6	(2848.0–6560.1) ^a^	4466.3	(3139.6–6285.3) ^a,b^	17.9	<0.01
Potassium (mg)	2462.1	(1627.4–3312.2) ^c,d^	2412.3	(1784.8–3269.5) ^c,d^	2989.0	(2143.0–4305.9) ^a,b^	2780.6	(2031.3–3750.6) ^a,b^	36.9	<0.01
Thiamine (mg)	0.93	(0.7–1.4) ^c,d^	0.94	(0.7–1.3) ^c,d^	1.07	(0.8–1.6) ^a,b^	1.05	(0.8–1.4) ^a,b^	30.6	<0.01
Riboflavin (mg)	0.80	(0.6–1.2) ^c^	0.79	(0.5–1.1) ^c,d^	1.04	(0.7–1.5) ^a,b^	0.89	(0.6–1.3) ^b^	31.7	<0.01
Niacin (mg)	11.5	(8.4–17.3) ^c,d^	12.2	(9.1–16.9) ^c,d^	14.9	(10.9–19.8) ^a,b^	13.8	(10.4–19.6) ^a,b^	35.6	<0.01
Vitamin A (μgRE)	428.1	(216.5–776.6) ^c^	388.4	(197.8–780.6) ^c,d^	632.7	(351.8–1032.0) ^a,b^	487.7	(257.1–889.1) ^b^	28.5	<0.01
Vitamin C (mg)	78.5	(32.9–121.3)	64.4	(35.2–104.3) ^c,d^	88.3	(55.1–147.1) ^b^	74.5	(45.9–121.7) ^b^	19.6	<0.01
^†^ Comparison of food intake over 24 h with usual intake (yes, *n* (%))	Ate more than usual	12 (9.5)	24 (5.2)		11 (8.1)		31 (5.6)			0.40
	Ate the same as usual	107 (84.9)	417 (91.0)		116 (85.9)		503 (91.1)			
	Ate less than usual	7 (5.6)	17 (3.7)		8 (5.9)		17 (3.1)			
**Women**										
**Variables (Unit)**	**Sarcopenic diabetes ^a^** **(*n* = 71)**		**Sarcopenia alone ^b^** **(*n* = 312)**		**Diabetes alone ^c^** **(*n* = 249)**		**Non-sarcopenia** **and non-diabetes ^d^** **(*n* = 1049)**		**K-W test**	
	**Median**	**(IQR: Q1–Q3)**	**Median**	**(IQR: Q1–Q3)**	**Median**	**(IQR: Q1–Q3)**	**Median**	**(IQR: Q1–Q3)**	***H*-value**	***p*-value**
Total energy (kcal)	1274.3	(1072.1–1609.6)	1273.5	(975.4–1590.6) ^c,d^	1418.0	(1125.1–1746.6) ^b^	1411.2	(1109.1–1774.7) ^b^	27.9	<0.01
Carbohydrate (g)	248.1	(206.8–310.3)	242.2	(186.8–304.0) ^c,d^	279.5	(212.5–340.2) ^b^	277.4	(214.7–343.6) ^b^	35.1	<0.01
Protein (g)	37.3	(26.0–51.1)	37.0	(26.9–52.7) ^c,d^	43.0	(31.6–57.1) ^b^	42.0	(30.2–56.4) ^b^	16.7	<0.01
Fat (g)	11.3	(7.2–20.8)	12.6	(7.9–21.9)	14.7	(8.7–21.4)	13.6	(7.7–22.6)	2.8	0.42
Crude Fiber (g)	4.76	(3.4–7.4)	4.29	(2.8–6.9) ^c,d^	5.50	(3.6–8.7) ^b^	5.25	(3.4–8.1) ^b^	24.1	<0.01
Calcium (mg)	260.1	(144.6–451.2)	272.5	(153.4–437.5) ^c^	314.9	(189.6–523.0) ^b^	292.8	(176.1–449.7)	10.1	0.02
Phosphorus (mg)	738.1	(576.0–1002.8)	713.3	(527.5–985.8) ^c,d^	823.3	(627.0–1060.6) ^b^	809.9	(604.1–1047.7) ^b^	17.8	<0.01
Iron (mg)	7.9	(4.7–12.5)	7.5	(4.7–12.0) ^c,d^	9.8	(6.2–16.2) ^b^	8.7	(5.6–13.9) ^b^	21.2	<0.01
Sodium (mg)	2364.9	(1442.4–3450.6) ^c,d^	2605.3	(1581.6–3905.1) ^c,d^	2963.5	(2024.5–4377.5) ^a,b^	2895.2	(1876.1–4451.9) ^a,b^	17.6	<0.01
Potassium (mg)	1850.4	(1366.3–2517.5)	1754.1	(1170.9–2514.5) ^c,d^	2008.1	(1614.4–2986.4) ^b^	2064.6	(1413.1–2900.5) ^b^	26.2	<0.01
Thiamine (mg)	0.66	(0.5–0.8)	0.67	(0.5–1.0) ^c,d^	0.75	(0.6–1.0) ^b^	0.77	(0.6–1.1) ^b^	20.6	<0.01
Riboflavin (mg)	0.50	(0.3–0.8)	0.58	(0.3–0.9) ^c^	0.69	(0.5–1.0) ^b^	0.63	(0.4–0.9)	12.3	<0.01
Niacin (mg)	8.4	(6.5–11.1)	8.2	(6.0–12.0) ^c,d^	9.8	(7.3–13.6) ^b^	9.7	(7.0–13.2) ^b^	23.0	<0.01
Vitamin A (μgRE)	268.0	(132.4–599.4)	330.1	(137.6–654.3) ^c^	410.7	(204.6–819.2) ^b^	357.2	(151.5–685.6)	9.3	0.03
Vitamin C (mg)	50.0	(27.1–69.3)	48.9	(24.2–83.1) ^c,d^	60.2	(28.1–98.4) ^b^	60.4	(31.0–104.1) ^b^	19.4	<0.01
^†^ Comparison of food intake over 24 h with usual intake (yes, *n* (%))	Ate more than usual	4 (5.6)	13 (4.2)		15 (6.0)		80 (7.6)			0.31
	Ate the same as usual	61 (85.9)	282 (90.4)		210 (84.3)		887 (84.6)			
	Ate less than usual	6 (8.5)	16 (5.1)		24 (9.6)		80 (7.6)			

Note: *p*-values from the Kruskal–Wallis (K-W) test and ^†^ Chi-square test. Significantly different (*p* < 0.05) from the: ^a^ Sarcopenic diabetes group, ^b^ Sarcopenia alone group, ^c^ Diabetes alone group, and ^d^ Non-sarcopenia and non-diabetes group. Post hoc tests for multiple pairwise comparisons between each group utilized K–W analysis with Dunn–Bonferroni correction and Chi-square test with Bonferroni correction. IQR: interquartile range, Q: quartile (Q1: 25th percentile, Q3: 75th percentile), *n*: number of participants.

**Table 4:** Comparison of practice frequency according to physical activity type and intensity of physical activity.

Variables	Men				*p*-Value
	Sarcopenic Diabetes ^a^ (*n* = 126)	Sarcopenia Alone ^b^ (*n* = 458)	Diabetes Alone ^c^ (*n* = 135)	Non-Sarcopenia and Non-Diabetes ^d^ (*n* = 552)	
	*n* (%)	*n* (%)	*n* (%)	*n* (%)	
Flexibility exercise ≥ 2 days/week (yes)	48 (38.1)	160 (34.9) ^d^	60 (44.4)	238 (43.1) ^b^	0.04
Resistance training ≥ 2 days/week (yes)	26 (20.6)	87 (19.0) ^c,d^	43 (31.9) ^b^	155 (28.1) ^b^	<0.01
Moderate-intensity physical activity ≥ 150 min/week (yes)	29 (23.0)	109 (23.8)	38 (28.1)	161 (29.2)	0.19
Vigorous-intensity physical activity ≥ 75 min/week (yes)	11 (8.7) ^c,d^	74 (16.2) ^c^	40 (29.6) ^a,b^	107 (19.4) ^a^	<0.01
**Variables**	**Women**				***p*-value**
	**Sarcopenic diabetes ^a^** **(*n* = 71)**	**Sarcopenia alone ^b^** **(*n* = 312)**	**Diabetes alone ^c^** **(*n* = 249)**	**Non-sarcopenia** **and non-diabetes ^d^** **(*n* = 1049)**	
	***n* (%)**	***n* (%)**	***n* (%)**	***n* (%)**	
Flexibility exercise ≥ 2 days/week (yes)	17 (23.9)	98 (31.4)	89 (35.7)	311 (29.6)	0.16
Resistance training ≥ 2 days/week (yes)	7 (9.9)	20 (6.4)	15 (6.0)	82 (7.8)	0.57
Moderate-intensity physical activity ≥ 150 min/week (yes)	5 (7.0) ^d^	58 (18.6)	51(20.5)	249 (23.7) ^a^	<0.01
Vigorous-intensity physical activity ≥ 75 min/week (yes)	5 (7.0)	34 (10.9)	29 (11.6)	149 (14.2)	0.16

Note: *p*-values are from the Chi-square test. Significantly different (*p* < 0.05) from the: ^a^ Sarcopenic diabetes group, ^b^ Sarcopenia alone group, ^c^ Diabetes alone group, and ^d^ Non-sarcopenia and non-diabetes group. Bonferroni post hoc analyses were used to identify any group difference. Each physical activity was set to 1 if it met the practice criteria, and 0 otherwise, *n*: number of participants.

**Table 5:** Association of sarcopenia and diabetes with physical inactivity.

		Crude OR	(95% CI)	*p*-Value	Model 1 OR ^†^	(95% CI)	*p*-Value	Model 2 OR ^‡^	(95% CI)	*p*-Value
Vigorous-intensity physical inactivity										
Men	Non-sarcopenia and non-diabetes	1.00 (reference)								
	Sarcopenic diabetes	2.87	(1.54–5.37)	<0.01	2.78	(1.47–5.33)	<0.01	2.68	(1.42–5.19)	<0.01
	Sarcopenia alone	1.43	(1.00–1.85)	0.03	1.30	(0.86–1.82)	0.17	1.25	(0.83–1.76)	0.25
	Diabetes alone	0.65	(0.43–0.98)	0.04	0.66	(0.44–1.01)	0.06	0.66	(0.43–1.01)	0.05
Women	Non-sarcopenia and non-diabetes	1.00 (reference)								
	Sarcopenic diabetes	2.15	(0.87–4.26)	0.08	1.96	(0.78–3.89)	0.13	1.93	(0.76–3.84)	0.14
	Sarcopenia alone	1.39	(0.97–2.05)	0.08	1.38	(0.92–2.04)	0.11	1.36	(0.91–2.03)	0.13
	Diabetes alone	1.35	(0.90–2.00)	0.15	1.35	(0.91–2.04)	0.15	1.35	(0.90–2.04)	0.15
Moderate-intensity physical inactivity										
Men	Non-sarcopenia and non-diabetes	1.00 (reference)								
	Sarcopenic diabetes	1.52	(1.02–2.34)	0.05	1.70	(1.11–2.66)	0.02	1.61	(1.06–2.55)	0.03
	Sarcopenia alone	1.39	(1.05–1.76)	0.01	1.57	(1.12–2.09)	<0.01	1.49	(1.06–1.99)	0.02
	Diabetes alone	1.01	(0.70–1.51)	0.96	0.99	(0.68–1.49)	0.97	0.98	(0.67–1.47)	0.90
Women	Non-sarcopenia and non-diabetes	1.00 (reference)								
	Sarcopenic diabetes	3.52	(1.91–7.39)	<0.01	3.63	(1.93–7.62)	<0.01	3.58	(1.91–7.56)	<0.01
	Sarcopenia alone	1.34	(1.02–1.74)	0.04	1.40	(1.02–1.84)	0.03	1.37	(1.01–1.81)	0.04
	Diabetes alone	1.09	(0.79–1.40)	0.54	1.07	(0.77–1.38)	0.67	1.07	(0.77–1.38)	0.67
Not walking										
Men	Non-sarcopenia and non-diabetes	1.00 (reference)								
	Sarcopenic diabetes	0.91	(0.54–1.57)	0.74	0.94	(0.55–1.68)	0.83	0.91	(0.53–1.64)	0.75
	Sarcopenia alone	0.85	(0.62–1.23)	0.35	0.85	(0.59–1.36)	0.44	0.82	(0.57–1.33)	0.37
	Diabetes alone	0.50	(0.30–1.02)	0.03	0.51	(0.30–1.03)	0.04	0.50	(0.30–1.03)	0.04
Women	Non-sarcopenia and non-diabetes	1.00 (reference)								
	Sarcopenic diabetes	1.99	(1.34–3.54)	<0.01	1.69	(1.14–3.18)	0.05	1.70	(1.14–3.19)	0.05
	Sarcopenia alone	0.82	(0.60–1.13)	0.22	0.76	(0.55–1.08)	0.12	0.77	(0.55–1.09)	0.13
	Diabetes alone	1.09	(0.83–1.57)	0.59	1.11	(0.84–1.62)	0.55	1.11	(0.84–1.62)	0.55
<600 METs										
Men	Non-sarcopenia and non-diabetes	1.00 (reference)								
	Sarcopenic diabetes	1.70	(1.14–2.61)	0.01	1.69	(1.14–2.71)	0.02	1.61	(1.08–2.58)	0.03
	Sarcopenia alone	1.41	(1.05–1.83)	0.02	1.37	(0.99–1.95)	0.07	1.30	(0.94–1.85)	0.13
	Diabetes alone	0.86	(0.56–1.39)	0.51	0.87	(0.56–1.40)	0.56	0.85	(0.55–1.38)	0.51
Women	Non-sarcopenia and non-diabetes	1.00 (reference)								
	Sarcopenic diabetes	2.48	(1.58–4.15)	<0.01	2.41	(1.53–4.15)	<0.01	2.37	(1.50–4.07)	<0.01
	Sarcopenia alone	1.09	(0.83–1.38)	0.52	1.14	(0.86–1.49)	0.35	1.12	(0.84–1.46)	0.43
	Diabetes alone	1.17	(0.87–1.50)	0.28	1.13	(0.83–1.46)	0.41	1.12	(0.83–1.46)	0.43

Note: *p*-values are from the binomial logistic regression analysis. OR: odds ratio, 95% CI: 95% confidence interval, MET: metabolic equivalent of task, ^†^ Model 1: Adjusted for age, body mass index, and sleep duration, ^‡^ Model 2: Adjusted for age, body mass index, sleep duration, drinking, and total energy intake. Each physical activity was set to 1 (inactive) if not practiced, and 0 (active) if practiced.

## Data Availability

All data generated or analyzed during this study are included in this published article. In addition, upon reasonable request, data supporting the findings of this study are provided by the corresponding author.
